# Prevalence and Genetic Characterization of *Cryptosporidium* Spp. In Diarrheic Children from Gonbad Kavoos City, Iran

**Published:** 2015

**Authors:** Mitra SHARBATKHORI, Ehsan NAZEMALHOSSEINI MOJARAD, Niloofar TAGHIPOUR, Abdol Sattar PAGHEH, Fatemeh MESGARIAN

**Affiliations:** 1 *Laboratory Science Research Center, Golestan University of Medical Sciences, Gorgan, Iran*; 2 *Dept. of Parasitology and Mycology, School of Medicine, Golestan University of Medical Sciences, Gorgan, Iran*; 3 *Gastroenterology and Liver Diseases Research Center, Shahid Beheshti University of Medical Sciences, Tehran, Iran*; 4 *Toxoplasmosis Research Center, Mazandaran University of Medical Sciences, Sari, Iran*; 5 *Laboratory of Gonbad Health Center, Golestan University of Medical Sciences, Gorgan, Iran*

**Keywords:** *Cryptosporidium*, Subtypes, *Gp60* gene, Children, Iran

## Abstract

***Background:*** *Cryptosporidium* is an intestinal protozean parasite causing waterborne and foodborne outbreaks of diarrheal diseases. The present study was performed in order to find prevalence and subtypes of *Cryptosporidium* among children with diarrhea in Gonbad Kavoos City, Northern Iran.

***Methods:*** Diarrheic samples were collected from 547 children. The initial parasitological diagnosis was made based on detection of oocysts using the modified Ziehl-Neelsen acid-fast staining method. The positive microscopically samples were selected for sequence analysis of partial 60 kDa glycoprotein (*gp60*) gene.

***Results:*** Out of 547 collected samples, 27 (4.94%) were positive for *Cryptosporidium *oocysts. Fifteen from 27 positive samples successfully amplified in PCR. Sequences analysis of *gp60* gene in 15 *Cryptosporidium* isolates revealed that all of them (100%) were *C. parvum*. The results showed three subtypes of IIa subtype family (7 cases) including IIaA16G2R1, IIaA17G1R1, IIaA22G3R1 and one subtype of IId subtype family (8 cases). The most common allele was IId A17G1d (53.3%).

***Conclusion:*** The predominance of zoonotic subtype families of *C. parvum* species (IIa, IId) in the present study is in concordance with previous studies in Iran and emphasizes the significance of zoonotic transmission of cryptosporidiosis in the country.

## Introduction

Cryptosporidiosis is one of the most important zoonotic protozoan diseases caused by *Cryptosporidium* spp. The organism has a wide host range that includes humans and domestic animals throughout the world. Transmission of infection can be occurred by ingesting oocysts of the parasite thorough the fecal oral route. Many vertebrates, including human, are affected by pathologic changes created by this parasite (1).

*Cryptosporidium *spp. is a main pathogen causing acute diarrhea, nonspecific signs such as dehydration, anorexia, fever, and weakness. Diarrhea is generally self-limiting in immunocompetent persons. However, it may be major public health importance in children as well as in immunocompromised people (2). Molecular biology has established powerful new tools for categorizing *Cryptosporidium *and has revealed significant variation within the genus. Currently, the genus *Cryptosporidium* consists of 30 species. *C. parvum* and *C. hominis *are two species predominantly found in humans. However, other species such as *C. meleagridis*, *C. muris*,* C. felis*, *C. canis*, *C. suis* and *C. andersoni* have been occasionally detected in feces of immunocompetent and immunocompromised individuals. Recently, sequencing data of 60 KDa glycoprotein (*gp60*) gene have revealed substantial genetic heterogeneity among *C. hominis *and *C. parvum* isolates establishing different subtype families within both species including Ia, Ib, Id, Ie, If and Ig for *C. hominis* and IIa, IIb, IIc, IId, IIe, IIf , IIg, IIh, IIi, IIk, and IIl for *C. parvum* (3, 4). 

As there is no genetic data about *Cryptosporidium* isolates in Gonbad Kavoos City, Northern Iran, this study aimed to find prevalence and identify the subtypes of the *Cryptosporidium* isolates from children with diarrhea using sequence analysis of the partial *gp60* gene in this region.

## Materials and Methods

***Study population***

The study was performed between November 2011 and October 2012 in two hospitals, namely Social Security and Taleghani hospitals in Gonbad kavoos City located in Golestan Province, Northern Iran, south eastern the Caspian Sea. A total of 547 children with diarrhea were examined for this study.

***Collection of samples***

Stool specimens were collected from each child. An informed consent was obtained from one of children's parents. The samples were concentrated by formalin-ethyl-acetate sedimentation method and stained using the modified Ziehl-Neelsen technique to detection of cryptosporidiosis. Aliquots of *Cryptosporidium* oocysts positive samples were preserved in 2.5% potassium dichromate and kept at 4 °C until DNA extraction.

***DNA extraction***

Genomic DNA was extracted from oosysts positive stool samples after washing three times with distilled water to removing the potassium dichromate. The QIAamp® DNA Stool Kit (Qiagen, Hilden, Germany) was employed for DNA extraction according to the manufacturer's instructions. The extracted DNA was stored at -20 °C until PCR analysis.

***gp60*** **Nested Polymerase chain reaction and sequencing**

A fragment of 400-500 bp within the *gp60* gene (*gp60*) was amplified by nested PCR from genomic DNA samples as described previously (5). PCR was conducted in 20 µL volumes using 10 pmol of each primer, 200 µM of each dNTP, 2mM MgCl2, and 1U Taq DNA Polymerase (Cinnagen, Tehran, Iran) ,1–10 ng of template DNA using a Techne TC-412 Thermal Cycler (Bibby Scientific Limited, Staffordshire, UK). The following primers were used in the first round of PCR: 5′-ATA GTC TCC GCT GTA TTC-3′ and 5′-GCA GAG GAA CCA GCA TC-3′ employing the following cycling protocol: one cycle at 94 °C for 3 min (initial denaturation), followed by 32 cycles of 94° C for 30 s (denaturation), 42 °C for 30 s (annealing), and 72 °C for 1 min (extension), followed by a final extension at 72 °C for 7 min. In the second round, 1 µL of the primary amplicon was subjected to the PCR using primers 5′-TCC GCT GTA TTC TCA GCC-3′ and 5′-GAG ATATAT CTT GGT GCG -3′ employing the same cycling protocol. A known *C. parvum* and a sample without DNA were included in each set of PCR as positive and negative controls, respectively. 

The secondary PCR products were sequenced using a BigDye Terminator Cycle Sequencing Kit (Applied Biosystems, Foster City, CA) in a Genetic Analyzer PrismTM 3130x1 (Applied Biosystems, Foster City, CA). All sequences were analyzed and compared with each other and previously reported sequences for identification of the alleles and subtypes reference sequences using the Chromas software (v. 2.4). 

## Results

**Fig. 1:**
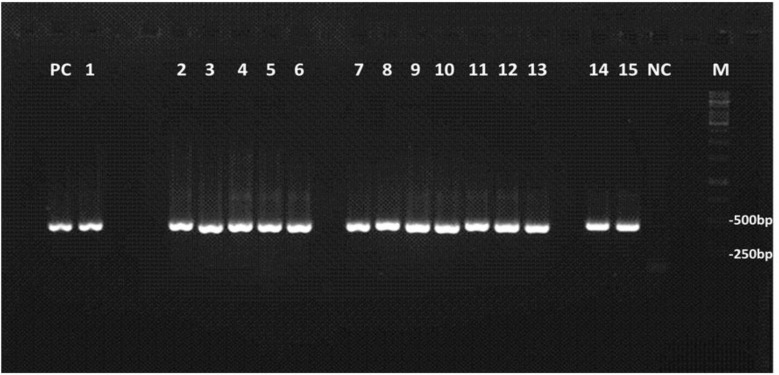
PCR of *Cryptosporidium *isolates from Iranian children based on partial *gp60 *gene belonged to *C. parvum*. Lanes 1-15 *Cryptospoidium* isolates, PC: positive control, NC: negative control, M: DNA size marker

Out of 547 collected diarrheic samples from children, *Cryptosporidium* oocysts were found in 27 (4.9%) using the modified Ziehl-Neelsen technique. The PCR amplicons about 400 base pairs of *gp60* gene were successfully obtained for 15 (of 27) *Cryptosporidium *positive cases on gel electrophoresis (Fig. 1). Sequence analyses of the partial *gp60* sequence data, using well-defined reference sequences for comparison, allowed the genotypic and subgenotypic classification of isolates. Subtypes were identified according to the number of trinucleotide repeats (TCA or TCG) coding for the amino acid serine (6). All isolates were *C. parvum *species. Four subtypes within two subtype families were identified. Seven (of 15) isolates belonged to the subtype family IIa and remaining 8 isolates belonged to IId. Three subtypes were recognized within the subtype family IIa including IIaA16G2R1 (2/15), IIaA17G1R1 (1/15), IIaA22G3R1 (4/15) while IIdA17G1d (8/15) was the only subtype within IId subtype family (Table 1). Anthroponotic subtype family IIc was not observed among isolates. Four representative sequences of *C. parvum* subtypes obtained from 15 human isolate in this study submitted to the GenBank under accession numbers: KM114269 to KM114272.

**Table 1:** Distribution of *Cryptosporidium parvum* subtypes in isolates from Iranian diarrheic children from Gonbad Kavoos City, Northern Iran

**Subtype**	**No. of ** **isolates**	**Accession ** **numbers**
IIaA16G2R1	2	KM114269
IIaA17G1R1	1	KM114270
IIaA22G3R1	4	KM114271
IIdA17G1d	8	KM114272

## Discussion

Various prevalences have been described for cryptosporidiosis from different parts of Iran. In this study, a prevalence rate of 4.94% (27/547) was obtained for cryptosporidiosis among diarrheic children from Gonbad Kavoos City, northern Iran. 

Similar prevalence has been described from Isfahan (4.6%) the country among diarrheic children (7). Lower prevalence have been reported from Mazandaran Province (0 and 2.3%) in the north, and central provinces of Tehran and Qazvin (1.1%, 2.5%) (8-11). However, higher prevalence has been stated from West Azerbayjan (7.66%) in the Northwest and Bandarabbas (7%) and Shiraz (25.6%) southern the country (12-14). An extensive range of prevalence of the disease have also been described in diarrheic children from other countries, including 0.9% in Malaysia, 1.9% in Philippine, 3.4% in Kuwait, 18.9% in Iraq, 25.3% in Uganda (15-18). The type of technique used for diagnosis and geographical area has been stated to affect the prevalence of cryptosporidiosis (19). Furthermore, dissemination of the parasite in each community and country depends on extent of contamination of water and food, animal contact, health measurements etc.

Previous studies have identified both *C. parvum* and *C.* *hominis* in human, with *C. parvum* as the predominant species responsible for human cryptosporidiosis in Iran (8, 20-23). 

In this study, using sequence analysis of partial *gp60* (p*gp60*) gene, all *Cryptosporidium* isolates (100%) from diarrheic children were identified as *C. parvum* species and none of them belonged to *C. hominis*. That emphasizes the importance of zoonotic transmission of cryptosporidiosis in the country. The predominance of *C. parvum* species in human cryptosporidiosis in Iran is consistent with studies from some developing and developed countries such as Malaysia, Kuwait, Yemen, Sweden, United Kingdom, Netherland, France, Portugal, Nicaragua (6, 24-31). In contrast, the predominance of *C. hominis* in human isolates have been reported from Australia (80.2%), India (75%), Egypt (60.5%), Mexico (83.33%) and Peru (70%) (32-36).

Other *Cryptosporidium* species reported to infect human such as *C. meleagridis*, *C. muris*,* C. felis*, *C. canis*, and *C. andersoni* were not found in the present study (37). 

Some of *C. parvum* subtype families such as IIa and IId, are found in both human and livestock responsible for zoonotic transmission of cryptosporidiosis. IId is a major zoonotic subtype family reported in Europe, Asia, Egypt and Australia (38). Eight of fifteen (53.3%) isolates in our study belonged to this subtype family. IIdA17G1d was the most common subtype and the only subtype within IId subtype family. This subtype has been reported in calves from Sweden (39). IIa is the predominant subtype family in animals and human worldwide (38). Seven of fifteen (46.7%) isolates in the current study belonged to IIa subtype family. The second common subtype was IIaA22G3R1 (4/15). This subtype previously has been reported in human isolates from Australia (40, 41). Two isolates were identified as subtype IIaA16G2R1. This subtype previously has been identified in calves from Poland, Netherland, Belgium, France, Spain, Portugal, United States and Canada (42-45). In addition, this subtype has been found in a sample of UV treated water from Portugal (46). Subtype IIaA17G1R1 was identified in one isolate that have been described in human isolates from England and Sweden (30, 47). This subtype has been extensively reported in calves from European countries and Argentina (45, 48). Recently, this subtype has been found in Romanian newborn lambs (49). 

The predominance of subtype families IId and IIa and lack of anthroponotic IIc subtype family in the current study is in a close agreement with few subtype analysis studies of cryptosporidiosis that have been performed using sequence analysis of p*gp60* gene in Iran. In the study of Nazemalhosseini-Mojarad et al. 36.4% (8/22) and 63.6% (14/22) of human isolates belonged to IIa and IId subtype family, respectively and they did not report any other subtype family (21). In Tehran, 58% (11/19) human isolates were IId subtype family and 31.5% (6/19) were IIa subtype family and 10.5% (2/19) belonged to If subtype family (22). In the current study subtypes IIaA16G2R1, IIaA17G1R1, IIaA22G3R1 and IIdA17G1d are reported for the first time in Iran.

## Conclusion

*C. parvum* was the only species found in Iranian children suffering from diarrhea and other *Cryptosporidium* species reported to infect the human were not found here. Detecting the subtype families IIa and IId of *C. parvum* in children suggest that zoonotic transmission play a more important role in human Cryptosporidiosis in Iran. Larger scale studies on subtype analysis of *Cryptosporidium* isolates from human and domestic animals in other regions of Iran is needed to improve our knowledge of cryptosporidiosis transmission in the country.
